# Effects of Green Tea Supplementation on Elements, Total Antioxidants, Lipids, and Glucose Values in the Serum of Obese Patients

**DOI:** 10.1007/s12011-012-9448-z

**Published:** 2012-05-15

**Authors:** Joanna Suliburska, Pawel Bogdanski, Monika Szulinska, Marta Stepien, Danuta Pupek-Musialik, Anna Jablecka

**Affiliations:** 1 Department of Human Nutrition and Hygiene, Poznan University of Life Sciences, Wojska Polskiego 31 Str., 60-624 Poznan, Poland; 2 Department of Internal Medicine, Metabolic Disorders and Hypertension, Poznan University of Medical Sciences, Poznan, Poland; 3 Department of Clinical Pharmacology, Poznan University of Medical Sciences, Poznan, Poland

**Keywords:** Obesity, Green tea, Polyphenol epigallocatechin-3-gallate, Minerals, Oxidative stress, Lipid, Glucose

## Abstract

The consumption of green tea has been associated with cardiovascular and metabolic diseases. There have been some studies on the influence of green tea on the mineral status of obese subjects, but they have not yielded conclusive results. The aim of the present study is to examine the effects of green tea extract on the mineral, body mass, lipid profile, glucose, and antioxidant status of obese patients. A randomized, double-blind, placebo-controlled study was conducted. Forty-six obese patients were randomly assigned to receive either 379 mg of green tea extract, or a placebo, daily for 3 months. At baseline, and after 3 months of treatment, the anthropometric parameters, blood pressure, and total antioxidant status were assessed, as were the levels of plasma lipids, glucose, calcium, magnesium, iron, zinc, and copper. We found that 3 months of green tea extract supplementation resulted in decreases in body mass index, waist circumference, and levels of total cholesterol, low-density cholesterol, and triglyceride. Increases in total antioxidant level and in zinc concentration in serum were also observed. Glucose and iron levels were lower in the green tea extract group than in the control, although HDL-cholesterol and magnesium were higher in the green tea extract group than in the placebo group. At baseline, a positive correlation was found between calcium and body mass index, as was a negative correlation between copper and triglycerides. After 3 months, a positive correlation between iron and body mass index and between magnesium and HDL-cholesterol, as well as a negative correlation between magnesium and glucose, were observed. The present findings demonstrate that green tea influences the body's mineral status. Moreover, the results of this study confirm the beneficial effects of green tea extract supplementation on body mass index, lipid profile, and total antioxidant status in patients with obesity.

## Introduction

Overweight and obesity represent rapidly growing threats to the health of populations in an increasing numbers of countries worldwide. The adverse health consequences associated with obesity include cardiovascular disease, type 2 diabetes, hypertension, dyslipidemia, cancers, and respiratory problems. Increasing evidence shows that obesity is associated with insulin resistance, with chronic low-grade inflammatory responses, and with oxidative stress [1, 2].

Oxidative stress consists of unbalanced higher cellular levels of reactive oxygen species (ROS), e.g., superoxide and hydroxyl radicals and cellular antioxidant defense [3]. The generation of ROS is ubiquitous, since such species are generated during aerobic metabolism, i.e., during mitochondrial and glycated protein oxidations. Blood may be vulnerable to oxidative stress induced by diabetes and may become exposed to ROS continuously generated by the auto-oxidation of hemoglobin and polyunsaturated fatty acids (PUFAs) [4, 5]. Because of their high rate of oxygen consumption, their high content of PUFAs, and their poor enzymatic antioxidant defense, blood cells exhibit increased vulnerability to diabetes-induced oxidative stress [4, 5]. Lipid peroxidation (LP) causes injury to cells and to intracellular membranes and may lead to cell damage and subsequently to cell death [6]. In order to scavenge ROS, various enzymatic and nonenzymatic antioxidant defense systems exist in the blood [7].

Moreover, many of the metabolic imbalances involved in obesity (e.g., glycemic, lipidemic) give rise to organ dysfunction and accelerated aging processes [8]. Several studies show obesity-related abnormalities in mineral status. The association between overweight, obesity, Fe, calcium Ca, and Zn deficiency has been observed in clinical and epidemiological studies [9]. Several studies suggest that Ca may play a role in the regulation of abdominal fat mass in obese people [10].

Understanding these pathological processes is crucial for better prevention and treatment of patients with obesity. The possible use of so-called functional food in this group of patients is particularly intriguing.

In recent years, researchers throughout the world have been investigating the potential benefits of green tea (GT) and its most abundant catechin, polyphenol epigallocatechin-3-gallate (EGCG). The positive effects of GT supplementation have been observed particularly in the prevention and control of type 2 diabetes [11]. In recent years, there have been a number of trials in humans showing the favorable effects of GT supplementation on body weight and body composition [12]. GT also has positive effects in the prevention and treatment of cardiovascular diseases [13]. Human studies suggest that GT may contribute to a reduction in the risk of atherosclerosis and cancer, as well as to the promotion of anti-hypertensive effects, body-weight control, antibacterial effects, increases in bone-mineral density, anti-fibrotic properties, and neuroprotective power [14]. There is growing evidence showing the possibility of using GT in the prevention and treatment of obesity and coexisting diseases [11, 15].

Several reports have focused on the interactions of polyphenols with metal ions, e.g., Zn and Fe, and have suggested their possible influence on mineral distribution in the body [16, 17]. There are few studies on the effects of GT on mineral status. The small number of studies evaluating green tea extract's (GTE) influence on mineral status in obese patients have shown inconclusive results.

The aim of our study was to estimate the influence of a 3-month oral supplementation of GTE on serum concentrations of minerals, on body mass, on total antioxidant status, on the lipid profile, and on glucose concentration in obese patients.

## Materials and Methods

### Study Patients

Informed consent was obtained from all subjects, and the study was approved by the Research Ethics Committee of Poznan University of Medical Sciences (approval number 38/11). The research conformed to all the ethical requirements of the Helsinki declaration.

Among 145 registered patients with obesity screened at our outpatient clinic, a total of 46 (23 men, 23 women) were enrolled. The inclusion criteria were: age 30–60 years, body mass index (BMI) ≥ 30 kg/m^2^, stable body weight (less than 3 kg of self-reported change during the previous 3 months). The exclusion criteria were hypertension; diabetes; impaired glucose tolerance; a current need for pharmacological treatment; a history of coronary artery disease, stroke (including transient ischemic attack), congestive heart failure, or malignancy; a history of use of any dietary supplements within 3 months prior the study; abnormal liver, kidney or thyroid gland function; clinically significant inflammatory processes within the respiratory, digestive, or genitourinary tract, or in the oral cavity, pharynx, or paranasal sinuses; a history of infection in the month prior to the study; and any other condition that in the opinion of the investigator would make participation not in the best interest of the subject, or could prevent, limit, or confound the protocol-specified efficacy assessments.

### Study Design

A prospective, randomized, double-blind design was applied. Randomization was performed by an independent statistician. Both patients and investigators were blinded to randomization.

All patients were randomized to receive green tea extract (Olimp Labs, Poland) at one capsule per day, or a placebo, with the morning meal for 3 months. One capsule contains 379 mg green tea extract and 208 mg of EGCG. The quantity of minerals in one capsule of the supplement is shown in Table 1. On the basis of the level of minerals in the supplement, it can be concluded that the daily intake of minerals from green tea extract was negligible. The placebo consisted of pure microcrystalline cellulose. All subjects were instructed to maintain an isocaloric diet and to continue their previous eating habits during the study. Subjects did not change their diet or physical activity during the study. The exercise and daily eating regimes were comparable in both groups.

**Table 1:** The amount of minerals in green tea extract

Ca	Mg	Fe	Zn	Cu
μg/g				
61.12 ± 8.28	1098.04 ± 148.3	5.67 ± 0.34	1.81 ± 0.32	0.51 ± 0.17
μg/one capsule				
23.22 ± 3.42	416.24 ± 56.3	2.15 ± 0.06	0.68 ± 0.11	0.19 ± 0.03

At the baseline, and again after 3 months of treatment, the anthropometric parameters, blood pressure measurement, and other laboratory tests were performed on both groups.

### Anthropometric and Blood Pressure Measurements

Anthropometric measurements were conducted with patients wearing light clothing and no shoes. Weight was measured to the nearest 0.1 kg, and height to the nearest 0.5 cm. The BMI was calculated as weight divided by height squared (kilogram per square meter). Obesity was defined as BMI ≥ 30 kg/m^2^.

Resting seated blood pressure was measured three times, and an average value was calculated according to the guidelines of the European Society of Hypertension. Regular or large adult cuffs were used, depending on the patient's arm circumference.

### Biochemical Measurements

All participants had blood collected from a forearm vein in serum-separated tubes (without using an anticoagulant). The coagulated blood was left to clot at room temperature for 30 min, and then centrifuged for 15 min at 2,000 r.p.m. at 4 °C. The supernatant fluid was then separated. Blood samples were collected after an overnight fast and after 30 min in the supine position. Serum samples were stored at −20 °C for no longer than 2–3 days.

The iron, copper, zinc, calcium, and magnesium content in the serum was determined by flame atomic absorption spectrometry (AAS-3 spectrometer, Carl Zeiss, Germany with deuterium background correction). In order to obtain the concentrations of the serum bioelements, the samples were diluted (*v*/*v* 1:1) as follows: for Fe, Zn, and Cu analyses, 0.01 % Triton X-100 (Merck) was used, while for the Mg and Ca analysis, aqueous solutions consisting of 0.01 % Triton X-100 (Merck) and 0.05 % lanthanum chloride (Merck) were used. The content of Fe, Cu, Zn, Ca, and Mg in serum and urine samples was determined at the following wavelengths: 248.3 nm (Fe), 324.8 nm (Cu), 213.9 nm (Zn), 422.7 nm (Ca), and 285.2 nm (Mg). The accuracy of the method was verified by certified reference material (HUM ASY CONTROL 2 and URN ASY CONTROL 2, Randox) and reached 95 %, 99 %, 94 %, 99 %, and 102 % for Ca, Mg, Fe, Zn, and Cu, respectively.

Plasma total cholesterol (TC), high-density lipoprotein (HDL), triglycerides (TG), and fasting glucose were measured using commercial kits. Serum levels of lipids were assayed in the central laboratory of Poznan University Hospital. Low-density lipoprotein cholesterol (LDL-C) was calculated using Friedewald's formula. Levels of blood glucose were determined by the routine enzymatic method.

The accuracy and precision of the techniques used to assay the lipids and glucose were validated. Reproducibility was checked with control human serum. Accuracy was assessed through the recovery value and it ranges between 95 % and 109 % and the variability coefficient, as an indicator of method precision, did not exceed 10 %.

Total antioxidant status (TAS) was established with a Randox kit (*Randox laboratories, Crumlin, UK*). This method is based on ABTS (2.2 azinobis 3-ethybenzthiazolinesulfate) incubation with metmyoglobin, and H_2_O_2_ with eventual ABTS-derived radical formation. This radical has a stable blue-green color at a wave length of 600 nm. Antioxidants present in the sample weaken the color intensity in proportion to their concentration. Initial absorbance was recorded, and then after 3 min, was recorded again for each sample using a Specord M-40 Carl-Zeiss Jena spectrophotometer (*SPECORD M40, Carl Zeiss Jena, Germany*).

### Statistical Analysis

Detailed statistical analysis was performed using *Statistica for Windows.* The normality of the variables' distribution was verified using Shapiro–Wilk's test of normality. A logarithmic transformation was used to normalize non-normally distributed dependent variables. Mean values, standard deviation, and median were calculated. Baseline characteristics of biochemical and demographic variables and blood pressure values were compared for the GTE and placebo groups, using the standard *t* test for two groups. Paired *t* tests were utilized to examine within-group differences at baseline and after 3 months. The level of statistical significance was set to *p* < 0.05.

## Results

Forty-six out of 145 obese patients with hypertension screened at our outpatient clinic fulfilled the inclusion and exclusion criteria and were allocated equally into the GTE and placebo groups.

**Fig. 1:**
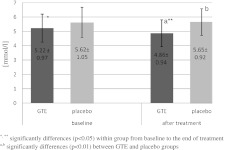
Concentration of total cholesterol (millimole per liter) in serum at baseline and after 3 months of treatment. ^*, **^ Significant differences (*p* < 0.05) within group from baseline to the end of treatment. ^a,b^ Significant differences (*p* < 0.01) between GTE and placebo groups

**Fig. 2:**
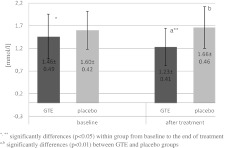
Concentration of triglycerides (millimole per liter) in serum at baseline and after 3 months of treatment. ^*, **^ Significant differences (*p* < 0.05) within group from baseline to the end of treatment. ^a,b^ Significant differences (*p* < 0.01) between GTE and placebo groups

The baseline characteristics of both groups are shown in Table 2, partly in Table 3, and in Figs. 1 and 2. There were no statistically significant differences (*p* > 0.05) between the two groups prior to the study.

**Table 2:** Baseline characteristic of both groups

Gender (male/female)	GTE group (*n* = 23)	Placebo (*n* = 23)	*p*
	11/12	12/11	NS
Age (years)	48.56 ± 8.81	52.26 ± 7.71	NS
Time since diagnosis of obesity (years)	9.70 ± 2.30	8.35 ± 2.85	NS
BMI (kg/m^2^)	32.07 ± 2.41	33.45 ± 2.65	NS
Waist circumference (cm)	101.78 ± 6.42	104.98 ± 6.35	NS

Comparison of GTE and placebo group with unpaired *t* test

Data are mean ± SD; *mean* the arithmetic mean; *SD* standard deviation, *n* number of subjects, *GTE* green tea extract, *BMI* body mass index, *NS* not significant, *p* level of significance

**Table 3:** Within-group anthropometric, blood pressure, and biochemical data at baseline and after 3 months of treatment

Variables	Baseline			After 3 months		
	GTE group (*n* = 23)	Placebo (*n* = 23)	*p*	GTE group (*n* = 23)	Placebo (*n* = 23)	*p*
BMI (kg/m^2^)	32.07 ± 2.41	33.45 ± 2.65	NS	31.71 ± 2.29	33.36 ± 2.66	0.03
Waist circumference (cm) SBP (mmHg)	101.78 ± 6.42 130.72 ± 6.95	104.98 ± 6.53 129.58 ± 7.88	NS NS	101.15 ± 6.42 128.15 ± 6.86	105.02 ± 6.10 128.32 ± 6.16	0.04 NS
DBP (mmHg)	85.10 ± 12.47	84.21 ± 3.32	NS	84.06 ± 12.26	84.50 ± 3.86	NS
LDL (mmol/L)	3.44 ± 1.03	3.80 ± 1.19	NS	3.02 ± 0.96*	3.71 ± 1.12	0.02
HDL (mmol/L)	1.12 ± 0.24	1.09 ± 0.25	NS	1.28 ± 0.26*	1.15 ± 0.21	NS
Glucose (mmol/L)	5.64 ± 0.44	5.67 ± 0.44	NS	5.48 ± 0.31*	5.59 ± 0.43	NS
TAS (mmol/L)	1.91 ± 0.23	1.90 ± 0.15	NS	2.09 ± 0.28*	1.91 ± 0.13	<0.01

Data are mean ± SD; the arithmetic mean; *SD* standard deviation, *n* number of subjects; *p < 0.05 from baseline to the end of treatment with paired *t* test

*GTE* green tea extract, *BMI* body mass index, *SBP* systolic blood pressure, *DBP* diastolic blood pressure, *LDL* LDL cholesterol concentration, *HDL* HDL cholesterol concentration, *TAS* total antioxidant status, *NS* not significant, *p* level of significance

### Between-Group Comparison after 3-Month Treatment

We found markedly lower levels of TC (*p* < 0.01), TG (*p* < 0.01), and LDL (*p* = 0.02) in the GTE group than in the control group after GTE treatment (Table 3, Figs. 1 and 2). Compared to the placebo group, BMI and waist circumference values significant decreased (respectively, *p* = 0.03 and *p* = 0.04) in GTE group after 3-month treatment (Table 3). TAS and Zn levels were markedly higher (respectively, *p* < 0.01 and *p* = 0.03) in the GTE group than in placebo group (Tables 3 and 4). No differences were found in the levels of glucose, HDL, blood pressure, or in the rest of the measured minerals between groups after treatment.

**Table 4:** Within-group minerals concentration in serum at baseline and after 3 months of treatment

Variables	Baseline			After 3 months		
	GTE group (*n* = 23)	Placebo (*n* = 23)	*p*	GTE group (*n* = 23)	Placebo (*n* = 23)	*p*
Fe (μmol/l)	17.91 ± 2.20	17.28 ± 3.16	NS	16.02 ± 3.19*	16.91 ± 2.97	NS
Zn (μmol/l)	12.09 ± 2.12	12.45 ± 3.11	NS	14.08 ± 3.16*	13.01 ± 2.98	<0.05
Cu (μmol/l)	13.97 ± 3.12	14.30 ± 2.13	NS	13.82 ± 2.98	14.45 ± 3.17	NS
Ca (mmol/l)	2.72 ± 0.59	2.75 ± 0.63	NS	2.92 ± 0.92	2.98 ± 0.69	NS
Mg (mmol/l)	0.97 ± 0.52	1.05 ± 0.76	NS	1.07 ± 0.68*	1.09 ± 0.43	NS

Data are mean ± SD; the arithmetic mean; *SD* standard deviation, *n* number of subjects; *p < 0.05 from baseline to the end of treatment with paired *t* test

*GTE* green tea extract, *NS* not significant, *p* level of significance

### Within-Group Comparison after 3-Month Treatment

Compared to the baseline measurements, 3-month treatment with GTE resulted in a change in the measured parameters only in the GTE group. GTE treatment caused significant reduction in TC and TG (respectively, *p* = 0.034 and *p* = 0.042) (Figs. 1 and 2), LDL (*p* = 0.035), glucose (*p* = 0.043), Fe (*p* = 0.048), and significant increase in HDL (*p* = 0.044), TAS (*p* = 0.036), Zn (*p* = 0.037), and Mg (*p* = 0.047)—see Tables 3 and 4.

### Correlation

A significant positive correlation between Ca and BMI (*p* = 0.042), and a negative correlation between Cu and TG (*p* = 0.038) were observed before treatment. After the 3-month supplementation, significant positive correlations between Fe and BMI (*p* = 0.023), and between Mg and HDL cholesterol (*p* = 0.023) were found, as was a significant negative correlation between Mg and glucose (*p* = 0.002) (Table 5).

**Table 5:** Significant correlation between minerals and other parameters in GTE group

Minerals	Parameters	*r*; *p*
Baseline		
Ca	BMI	0.33; 0.042
Cu	TG	−0.34; 0.038
After treatment		
Fe	BMI	0.37; 0.023
Mg	glucose	−0.48; 0.002
Mg	HDL	0.32; 0.023

*r*, Pearson's correlation coefficient; *p*, level of significance

## Discussion

The significant influence demonstrated in our study of a 3-month GTE supplementation on mineral levels in obese patients is a new finding. Moreover, the favorable effect of GTE supplementation on body mass, lipid profile, glucose, and antioxidant status was also demonstrated.

The potential of green tea as a natural agent of weight loss has been investigated in some other studies similar to our own [18, 19]. Some authors suggest that the polyphenolic components of green tea have an anti-obesogenic effect on fat homeostasis, by increasing thermogenesis, reducing fat absorption, and introducing modifications in appetite [20, 21]. The results obtained (significant differences in BMI between the GTE group and the placebo group, and the slight reduction in BMI in the group with GTE) support the possible role of green tea in weight loss.

A number of experimental studies have been conducted to examine the effects of GT on carbohydrate metabolism and lipid profile [15, 19, 22]. Wu et al. [15] demonstrated that during 12-week GT supplementation, insulin sensitivity in Sprague–Dawley rats increased. They concluded that the most active component in GT is polyphenol.

Abnormalities of lipid metabolism are one of the major risk factors for the development of cardiovascular disease, very often associated with obesity. The outcomes of our study show that a 3-month supplementation exerts a positive effect on lipid profile in patients with obesity. Decreases in TC, LDL, and TG concentration were observed, along with increases in HDL concentration (within the study group). The positive effect of GT in human studies has also been observed by others [19, 23]. The results could be partly caused by the decreased absorption of cholesterol and glucose from the diet in the presence of the polyphenols from GTE in the small intestine, especially if the supplement is taken by subjects during meals [24]. GT catechins influence micellar solubility, luminal lipid hydrolysis, and intestinal lipid absorption, and they may interfere with the emulsification, hydrolysis, and micellar solubilization of lipids, suggesting that the cholesterol-lowering effect of catechins may, at least in part, be mediated by their influence on intestinal lipid absorption [25, 26].

We found a beneficial influence of GTE on oxidative stress. An increase in TAS levels in the serum was observed after supplementation. Tea catechins eliminate reactive oxygen and nitric radicals, and also act as chelators of metal ions active in the system redox. Both radicals and active metal ions are highly toxic because of their destructive effect on lipids, proteins, and nucleic acid. They also interact to indirectly influence the inhibition of pro-oxidative enzymes, such as nitric oxide synthetase, lipooxygenase, cyclooxygenase, and xanthine oxidase [27, 28].

A literature review shows that the effect of GTE on mineral status has not been widely studied, and the present study shows novel results on this topic.

In our study, a decreased Fe level in the serum of obese patients supplemented with GTE was observed. This lower level of Fe in serum after treatment may be caused by the low availability of this mineral, as suggested by Zeyuan et al. [29]. In their experiment on rats, they found that GT and its water extract inhibited the absorption of Ca, Fe, and Zn, and promoted absorption of Cu. In particular, the polyphenols contained in GT chelate Fe and might cause it to be less well absorbed, leading to a lower concentration in the body [17, 30]. On the basis of our results, it can be concluded that GT supplementation may reduce the level of Fe in the blood of obese people, and thereby increase the deficit of this mineral in the body.

Moreover, in our study, an association was found between the concentration of Fe in serum and the BMI of patients after the 3-month treatment. Some authors have suggested that the alternation of Fe biomarkers in the obese population could be a result of obesity-related inflammation or of related comorbidities. Obesity has been reported to induce an inflammatory state, and some studies have suggested that inflammation contributes to high serum Fe levels in obese subjects [31]. In this study, the reduction in BMI index was associated with a decrease in inflammation biomarkers (unpublished data) and in the serum Fe level.

As with other authors, we found that GT treatment reduced Fe status and improved Zn and Mg status in patients and experimental animals [16, 17]. The increased Zn level in the serum may be associated with improved antioxidant status subsequent to GT treatment, as concluded by Hamdaoui et al. [16].

We have also observed an association between glucose and Mg, which confirms the results of other studies [32, 33]. In this study, we observed following supplementation that there were increased concentrations of Mg associated with decreased levels of glucose in the serum of the GTE group. It may be suggested that higher levels of Mg in the body led to a reduction in plasma glucose and stabilized the glucose level during treatment. Soltani et al. [34] concluded that Mg could improve the structure of the pancreas (especially in diabetic individuals) and may prevent morphological changes of blood vessels. In this study, the improvement in Mg status after GTE supplementation was associated with higher levels of HDL cholesterol in the serum of patients. A positive correlation between Mg in serum and HDL has also been observed by other authors [35]. The results obtained in experimental and clinical studies suggest the higher Mg levels in the body are associated with a beneficial effect on lipid profile [36, 37].

In this study, we did not find any change in Ca levels of serum during GTE treatment. Other studies have shown that supplementation with GT polyphenols results in an increase in bone-mineral density and a decrease in urinary calcium levels [38, 39]. In this study, we did not evaluate the concentration of this mineral in other tissues, such as bones. The lack of effect of GTE supplementation on serum Ca levels in patients in this study may be due to factors of homeostasis in the bodies of the subjects during treatment.

Moreover, at the baseline of the study, we observed a negative correlation between Cu and TG levels in serum. A similar association between Cu and TG in the serum of obese patients with hypertension was found in our previous study [40], and has also been observed by others [41]. These results confirm the association between Cu status and lipid profile in the body.

It is also worth noting that prior to the treatment, the patients' BMI was positively correlated with the concentration of Ca in serum. This result may be connected with the role of Ca in the regulation of abdominal fat mass in obese people [10].

## Conclusions

The present findings demonstrate strong evidence for the effect of GTE on the mineral status of obese people. Our results suggest that GT improves the status of Zn and Mg in such individuals. However, supplementation with GT can deepen the deficit of Fe which is observed in obesity. Moreover, this study confirms the beneficial influence of GTE supplementation on body mass, lipid profile, glucose, and TAS in obese patients. As the subjects represented in the studied group are overrepresented in the modern population, the potential advantages of GTE should be carefully evaluated. Further studies on a larger scale and with a longer time of observation are needed to support our data.
